# Potential Roles of Exosomal lncRNAs in the Intestinal Mucosal Immune Barrier

**DOI:** 10.1155/2021/7183136

**Published:** 2021-08-25

**Authors:** Shanshan Chen, Ruonan He, Beihui He, Li Xu, Shuo Zhang

**Affiliations:** The First Affiliated Hospital of Zhejiang Chinese Medical University, Hangzhou 310006, China

## Abstract

The intestinal mucosal immune barrier protects the host from the invasion of foreign pathogenic microorganisms. Immune cells and cytokines in the intestinal mucosa maintain local and systemic homeostasis by participating in natural and adaptive immunity. Deficiency of the intestinal mucosal immune barrier is associated with a variety of intestinal illnesses. Exosomes are phospholipid bilayer nanovesicles that allow cell-cell communication by secreting physiologically active substances including proteins, lipids, transcription factors, mRNAs, micro-RNAs (miRNAs), and long noncoding RNAs (lncRNAs). Exosomal lncRNAs are involved in immune cell differentiation and the modulation of the immune response. This review briefly introduces the potential role of exosomal lncRNAs in the intestinal mucosal immune barrier and discusses their relevance to intestinal illnesses.

## 1. Introduction

There are four types of intestinal mucosal barriers: mechanical, immunological, biological, and chemical. Impairment in any barrier causes an imbalance in intestinal mucosal immunity. The etiology of intestinal mucosal barrier dysfunction may independently or synergistically trigger a biochemical cascade leading to chronic inflammation and various intestinal diseases including inflammatory bowel disease (IBD), irritable bowel syndrome (IBS), and benign and malignant tumors of the colon [1]. The intestinal mucosal immune barrier is one of the most important protective barriers in the human body [2], since it maintains intestinal microflora balance which is crucial to resisting bacterial translocation, endotoxemia, and secondary damage [3].

Exosomes are extracellular phospholipid bilayer vesicles with an average diameter of 100 nm formed by the fusion of multivesicular bodies (MVBs) with the plasma membrane. Exosomes have donor cell-specific components including lipids, proteins, and nucleic acids (DNA, mRNA, miRNA, and lncRNA) that may be selectively absorbed by proximal and distal cells. These recipient cells may recode bioactive substances to influence cell physiological activities such as cell signal transduction, proliferation, apoptosis, differentiation, polarization, immune response, and antigen presentation [4, 5]. The exosomal pathway in the intestinal mucosal immune system is involved in maintaining homeostasis by promoting the function of the epithelial barrier and maintaining its integrity, inducing oral tolerance to harmless antigens, and exerting inhibitory effect of regulatory T (Treg) cells. The different exosomal functions suggest that the exosomal pathway may play a role in treating pathological intestinal inflammation [6].

lncRNAs are noncoding transcripts above 200 nucleotides which lack the structure of long reading frames [7, 8] and often exist as secondary stem loop-like structures or tertiary structures [9]. lncRNAs carried by exosomes are widely involved in intercellular material exchange and signal transduction. They are stable in biological fluids as endogenous RNA enzymes do not cause their degradation [10]. lncRNAs may act in the following ways: (1) bind to specific proteins to regulate their activities; (2) bind to specific proteins to change their cell localization; (3) act as the precursor molecule of small RNA (such as miRNA); (4) act as scaffolds to form nucleic acid-protein complexes; (5) modify the expression of downstream genes by mediating histone modification, chromatin remodelling, or inhibiting RNA polymerase II; (6) influence the expression of downstream genes by affecting transcription of the promoter; and (7) form complementary double strands with protein-coding gene transcripts to interfere with mRNA splicing or generating endogenous interfering RNA under the action of ribonuclease Dicer to inhibit gene expression [11, 12]. Exosomal lncRNAs regulate antigen presentation, affect the activity of immune cells, and induce apoptosis of related effector cells [13]. In summary, lncRNAs regulate gene expression at epigenetic, transcriptional, or posttranscriptional levels by interacting with proteins, DNA, and RNA and are involved in important processes such as intranuclear transport of chromatin, regulation of protooncogene activation, differentiation of immune cells, and regulation of the immune system [14].

A better understanding of the potential role of specific exosomal lncRNAs in the intestinal mucosal immune barrier may result in improved disease severity assessment and facilitate treatment with the help of biomarker lncRNAs.

## 2. Intestinal Mucosal Immune Barrier

The intestinal mucosal immune barrier arises to local and systemic immune reactions under the stimulation of intestinal antigens to protect the host from the damage. This barrier mainly comprises gut-associated lymphoid tissue (GALT), secretory immunoglobulin A (sIgA), and immune-producing substances such as cytokines [15]. GALT mainly includes Peyer's patches (PPs), isolated lymphoid follicles (ILFs), mesenteric lymph nodes (MLNs), and diffuse GALT. Diffuse GALT mainly includes intraepithelial lymphocytes (IELs) and lamina proprial lymphocytes (LPLs).

The immune function of the intestinal mucosal immune barrier is mainly exerted by the afferent induction sites (PP and ILF) and efferent effector sites (IEL and LPL). Immune cells in induced sites can take up, process, and present antigens, while the effector sites generate immune responses. Microfold cells (M cells) in PP combine with pathogen-associated molecular patterns (PAMPs) of intestinal pathogens through surface pathogen recognition receptors (PRRs); then, the enteric luminal antigens are endocytosed and transported to the follicular region for processing, which causes antigen-specific B- and T-lymphocyte excitation. M cells also transport antigens to the other side of the cell membrane and present antigens to antigen-presenting cells (APCs), which get transferred to the lamina propria and epithelium. Antigens entering the lamina propria and epithelium can stimulate the LPL and IEL of the effector sites and produce a series of immune responses.

The intestinal innate immune system is composed of mucus, the epithelial barrier, macrophages, monocytes, neutrophils, dendritic cells (DCs), natural killer (NK) cells, eosinophils, and basophils which nonspecifically recognize pathogens and maintain intestinal immune tolerance [16]. The innate immune response to pathogenic bacteria depends on the recognition of PAMPs by toll-like receptors (TLRs) which deliver risk signals to APCs. This causes the production of reactive oxygen species, nitrogen dioxide, proinflammatory factors and upregulates costimulatory molecule expression, which causes adaptive immune responses.

**Figure 1:**
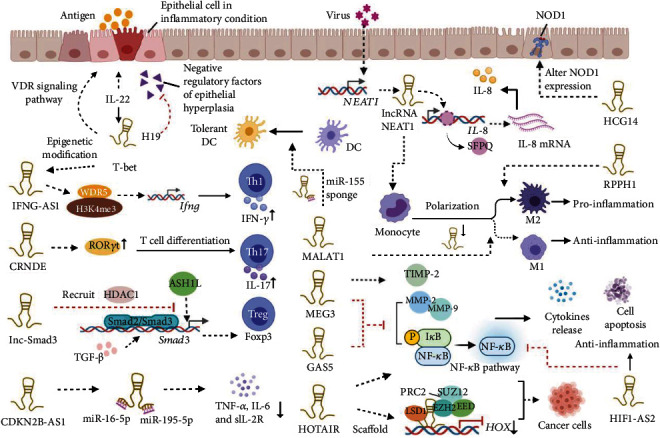
Functions of exosomal lncRNAs on intestinal mucosal immune system. Exosomal lncRNAs promote Th17 cell differentiation and IFN-*γ* secretion of Th1 cells, regulate the polarization of Treg cells and macrophages, reprogram DCs into a tolerant phenotype, affect the cytokine secretion and inflammatory signaling pathways, and participate in immune response and immune evasion of cancer cells.

IEL is a lymphocyte population mainly composed of CD8^+^ T cells that are the closest to antigens. They directly recognize unprocessed antigens and perform a variety of immune functions, including NK activity, specific cytotoxicity, and antitumor activity in the acquired immune system. They produce cytokines such as interleukin- (IL-) 2, IL-3, IL-4, IL-5, IL-10, interferon- (IFN-) *α*, and IFN-*γ* which regulate T helper (Th or CD4^+^) cells. LPL mainly includes CD4^+^ T cells and B cells. Naive CD4^+^ T cells may differentiate into various subgroups that are divided according to the different cytokines and effects they produce after being stimulated by external antigens, such as Th1, Th2, Th9, Th17, and Treg cells [17] (Figure 1).

## 3. Exosomal lncRNAs Regulate the Intestinal Mucosal Immune Barrier

### 3.1. Exosomal lncRNAs Regulate IBD

IBD is a chronic inflammatory disease of the gastrointestinal tract, including Crohn's disease (CD) and ulcerative colitis (UC). The chief clinical presentation includes abdominal pain, diarrhea, mucous bloody stool, and weight loss [18]. Immune dysregulation of IBD appears as an epithelial injury (abnormal mucus production and repair defect); inflammatory progression caused by intestinal flora; numerous inflammatory cell infiltrations in the lamina propria including T cells, B cells, macrophages, DCs, and neutrophils; and a failure of the immune system to regulate the inflammatory response [19–21]. Several studies have shown that exosomal lncRNAs participate in maintaining the intestinal mucosal immune barrier in IBD.

#### 3.1.1. H19

H19 from chromosome 11p15.5 is a 2.3 kb lncRNA involved in immune and inflammatory responses [22]. Its levels increase in inflammatory intestinal tissues of mice and human patients with UC [23]. H19 is an inflammatory lncRNA induced by IL-22, which antagonizes the negative regulatory factors of intestinal epithelial hyperplasia and suppresses p53 protein expression, and the miRNAs miR34a and let-7 [23]. It is an important intermediate signaling molecule which connects IL-22 signaling with other regulatory networks that control the repair of the intestinal epithelium under inflammatory conditions. An increase in H19 may be affiliated with the triggering of the mitogen-activated protein kinase (MAPK) pathway and apoptosis [24]. H19 may also deregulate the homeostatic role of Vitamin D receptor (VDR) signaling which is involved in regulating inflammation [25]. Increased H19 expression is correlated with decreased *VDR* expression observed in the colon biopsy of patients with UC [26]. Targeting the interaction between H19 and VDR receptor may lead to a cure for UC [26].

#### 3.1.2. IFNG-AS1

IFNG-AS1 (TMEVPG1 or NE ST) is a Th1-specific lncRNA mainly expressed in CD4^+^ T cells. It is correlated with increased expression and secretion of the inflammatory cytokine, IFN-*γ* [27], possibly due to its interaction with the WD repeat domain 5 (WDR5) subunit of the histone 3 lysine 4 (H3K4) methyltransferase complex [28]. IFN-*γ* is vital for maintaining intestinal mucosal immunity since it is an activator of phagocytes and neutrophils, an antagonist of IL-4, and promoter of T- and B-lymphocyte differentiation. IFNG-AS1 is associated with IBD susceptibility-related single-nucleotide polymorphism (SNP), rs7134599 [29]. IFNG-AS1 expression significantly increases in the intestinal mucosal tissues of IBD patients, 2,4,5-trinitrobenzenesulphonic acid- (TNBS-) induced colitis mouse models, and spontaneous colitis in IL-10 knockout mice [29]. IFNG-AS1 induces Th1 cell-specific expression of signal transducer and activator of transcription 4 (STAT4) and T-box expressed in T cells (T-bet) [30]. The epigenetic modification of T-bet on the proximal and distal enhancers of IFNG-AS1 may be related to IFN-*γ* expression in Th1 cells [28]. Expression of several upstream inflammatory regulators, including IFN-*γ*, IL-1, IL-6, and tumor necrosis factor-*α* (TNF-*α*), is elevated in UC patients, and these regulators enhance inflammatory responses in patients' Th1 cells. In summary, IFNG-AS1 may participate in the development of intestinal mucosal inflammation by regulating CD4^+^ T cell immune function in patients with IBD [27]. Its levels may be a marker to distinguish among active UC patients, remission patients, and healthy people.

#### 3.1.3. NEAT1

Nuclear paraspeckle assembly transcript 1 (NEAT1) is a nuclear-restricted lncRNA positioned on the subnuclear structure that is highly expressed in IBD and participates in several immune responses. TNF-*α* and dextran sulphate sodium (DSS) destroy the integrity of the intestinal epithelial barrier thereby leading to IBD development. Recent evidence shows that NEAT1 is involved in the inflammatory response by regulating the intestinal epithelial barrier and exosome-mediated polarization of macrophages in IBD [31]. M1 macrophages produce proinflammatory cytokines and aggravate the inflammatory process, while M2 macrophages are closely related to anti-inflammatory response and immune homeostasis. Downregulation or knockout of NEAT1 promotes macrophage M1 transformation to M2 and suppresses the inflammatory reactions [31]. IL-8 is produced by phagocytes and mesenchymal cells exposed to inflammatory stimuli. It activates neutrophils and participates in infection, inflammation, and ischemia. NEAT1 significantly affects the IL-8 levels by eliminating the inhibitory effect of splicing factor proline/glutamine-rich (SFPQ) protein on *IL-8* expression [32]. Additionally, NEAT1 knockout or inhibition blocks the release of inflammatory factors TNF-*α*, IL-1*β*, and IL-6 [33] resulting in the preservation of intestinal epithelial barrier integrity. Silencing NEAT1 inhibits advanced inflammatory factors vital for the activation, differentiation, and maturation of immune cells (IL-6, chemokine (C-X-C motif) ligand 5 (CXCL5), CXCL10, CXCL11, chemokine (C-C motif) ligand 2 (CCL2), and CCL8) which are induced via TLR2 signaling [34]. Meanwhile, activating TLR2 significantly induces NEAT1-V1 expression in THP-1 cells [34]. In summary, NEAT1 is a biomarker of IBD which induces inflammatory factors and evidence exists that inhibition of this lncRNA restores the intestinal epithelial barrier.

#### 3.1.4. GAS5

RNA-growth arrest-specific transcript 5 (GAS5) is an lncRNA located on chromosome 1q25.1 with many functions in inflammatory and autoimmune diseases such as rheumatoid arthritis (RA) and systemic lupus erythematosus (SLE) [35]. Its expression is significantly reduced in immune cells [36]. GAS5 inhibits gene expression by recruiting polycomb repressive complex 2 (PRC2) to the promoter of target genes [37]. Matrix metalloproteinases (MMPs) are proteolytic enzymes that participate in the damage and reconstruction of inflammation-related tissues by lysing components of the extracellular matrix (ECM), maintaining cell apoptosis, and promoting cytokine release [38]. Activated monocytes and macrophages are the major contributing cells of MMP2 and MMP9 in inflammatory diseases [39, 40], and these MMPs are highly expressed in colonic mucosa, serum, urine, and stool samples of patients with IBD [41–43]. GAS5 mediates intestinal mucosal damage since its overexpression and knockdown decrease and increase the levels of these MMPs, respectively [44]. GAS5 overexpression decreases the expression of TNF-*α*, IL-1*β*, IL-6, and IL-8. GAS5 is downregulated, and MMPs are upregulated in intestinal mucosal inflammatory tissues of patients with IBD [45]. Moreover, GAS5 adjusts lipopolysaccharide- (LPS-) induced inflammatory destruction by upregulating Kruppel-like factor 2 (KLF2) expression and inhibiting the nuclear factor kappa-light-chain-enhancer of activated B cells (NF-*κ*B) pathway [46]. It is evident that GAS5 upregulation has a protective effect on the intestinal mucosal immune barrier during the induction of inflammation.

#### 3.1.5. Other Exosomal lncRNAs Associated with IBD

The TLR signaling pathway leads to NF-*κ*B activation and increases the expression of proinflammatory factors which are involved in IBD pathogenesis [47]. The lncRNA hypoxia-inducible factor 1 antisense RNA-2 (HIF1A-AS2) protects the immune barrier and maintains intestinal immune homeostasis in UC patients by suppressing NF-*κ*B signaling pathway activation and inhibiting the upregulation of inflammatory factors [48]. The lncRNA CDKN2B-AS1 (antisense noncoding RNA in the INK4 locus (ANRIL)) regulates tight junction protein, and its level is negatively correlated with levels of inflammatory cytokines TNF-*α*, IL-6, and sIL-2R [49, 50]. CDKN2B-AS1 relieves inflammation of UC by sponging miR-195 and miR-16, providing an alternative for diagnosis and treatment of UC [50]. Colon rectal neoplasia differentially expressed (CRNDE) promotes DSS-induced apoptosis of colon epithelial cells by inhibiting miR-495 and increasing suppressor of cytokine signaling 1 (SOCS1), suggesting that CRNDE is a target for IBD treatment [51].

### 3.2. Exosomal lncRNAs Regulate Proliferation, Invasion, and Metastasis of Colorectal Cancer

Colorectal cancer (CRC) is the third most commonly diagnosed cancer with high mortality rates worldwide [52, 53]. The molecular mechanism of CRC remains unclear; however, exosomal lncRNAs are involved in the tumorigenesis and tumor metastasis as regulators of immune modulation [54]. Multiple inflammatory signaling pathways are present in tumor cells including the Janus kinase/signal transducers and activators of transcription (JAK/STAT), NF-*κ*B, and TLRs, which stimulate tumor cells proliferation, invasion, metastasis, and angiogenesis and suppress apoptosis when abnormally activated [55]. The Wnt/*β*-catenin signaling pathway may be involved in the inflammatory response by inhibiting or activating the NF-*κ*B signaling pathway [56, 57]. M2-type tumor-associated macrophages (TAMs) are the most numerous cells participating in tumorigenesis and play a prominent role in the progression of carcinogenesis to metastasis [58].

#### 3.2.1. HOTAIR

HOX transcript antisense intergenic RNA (HOTAIR) is a 2.3 kb noncoding region of chromosome 12q13.13, and the first known lncRNA with *trans*-transcriptional regulation. Its expression is much higher in CRC patients than that in healthy controls and is associated with high mortality [59]. HOTAIR knockdown and miR-203a-3p overexpression lead to inhibited CRC cell proliferation and reduced chemoresistance by suppressing the Wnt/*β*-catenin signaling pathway [60]. It also contributes to 5-fluorouracil resistance by inhibiting miR-218 and activating NF-*κ*B signaling pathway in CRC [61]. HOTAIR mainly acts as a scaffold to recruit and bind PRC2 and lysine-specific histone demethylase 1 complex (LSD1) to form a histone modification complex at the *Hox* gene site resulting in epigenetic silencing in the site, which promotes the development of malignant tumors [62]. The kernel components of the PRC2 complex are enhancers of zeste homolog 2 (EZH2), embryonic ectoderm development (EED), and suppressor of zeste 12 (SUZ12) [63]. The histone methyltransferase (EZH2) is the most critical subunit since it has an indispensable role in diverse cell types including immune cells [63]. Binding of EZH2 to the IL-4 promoter regulates Th1 and Th2 differentiation by inhibiting the transcription of granulocyte factors such as STAT6 and GATA3. EZH2 facilitates Treg cell differentiation and inhibits the differentiation of Th1, Th2, and Th17 cells [64]. EZH2 inhibition upregulates the expression of effector cytokines in CD4^+^T cells. Therefore, HOTAIR downregulation or inhibitors of HOTAIR-EZH2 may facilitate the immune response to reduce proliferation and invasion of CRC.

#### 3.2.2. CRNDE

Colon rectal neoplasia differentially expressed (CRNDE) is a 1 kb lncRNA located on chromosome 16q12.2. It is the protooncogene of CRC promoting the proliferation, migration, metastasis, and chemotherapy resistance of CRC [65, 66]. Tumor formation in colon triggers immune responses leading to IL-17-producing T cells, i.e., Th17 cells [67], which are closely linked with Treg cells, intestinal epithelial cells (IECs), APCs, and the intestinal flora to jointly maintain intestinal mucosal stability. ROR*γ*t promotes inflammation and differentiation of naive CD4^+^ T cells into Th17 cells by binding to *IL-17* promoter and inducing IL-17 secretion [68, 69]. E3 ubiquitin ligase Itch binds to the PPXY motif of ROR*γ*t and induces ubiquitination and degradation of ROR*γ*t [70]. The serum exosomal CRNDE-h level is positively correlated with the proportion of Th17 cells in the tumor-infiltrating T cells in CRC patients. CRNDE-h delivered by CRC exosomes is transmitted to CD4^+^ T cells and promotes Th17 cell differentiation by inhibiting the Itch-mediated ubiquitination and degradation of ROR*γ*t [71], which expands our understanding of Th17 cell differentiation in CRC.

#### 3.2.3. MALAT1

Metastase-associated lung adenocarcinoma transcript 1 (MALAT1) is an 8.7 kb lncRNA located on 11q13.1. It suppresses NF-*κ*B activity by binding with the p50/p65 heterodimer in the nucleus, thereby affecting the innate immune response [72]. Macrophage polarization is an important molecular event in the innate immune responses. Inhibiting MALAT1 expression curbs the polarization of M2-type macrophages and facilitates M1-type macrophage polarization [73]. MALAT1 inhibits the proliferation of mouse macrophage cell line (RAW264.7), while hsa-miR-346 promoted its proliferation [74] and induces DCs to become tolerant after LPS stimulation. Ectopic MALAT1 promotes the expression of DC-specific ICAM-3 grabbing nonintegrin (DC-SIGN) by acting as a sponge miR-155, which is important for the maintenance of DC tolerance [75]. Moreover, MALAT1 interacts with CCL5 to mediate the progression of CRC by tumor-associated DCs [76]. Resveratrol suppresses the invasion and migration of CRC by downregulating MALAT1 expression, resulting in decreased nuclear localization of *β*-catenin and reduced expression of downstream target genes c-Myc and MMP-7 [77].

#### 3.2.4. RPPH1

Ribonuclease P RNA component H1 (RPPH1) is located on chromosome 14q11.2 and mainly packaged in exosomes. It is upregulated in CRC specimens and is correlated with advanced tumor-node-metastasis (TNM) and poor prognosis [78]. It interacts with tubulin *β*3 class III (TUBB3) and is transmitted by exosomes to macrophages to mediate macrophage M2 polarization, thereby promoting CRC cell metastasis and proliferation. RPPH1 levels in plasma exosomes of CRC patients significantly decline after tumor removal [78], suggesting that RPPH1 downregulation inhibits the occurrence and development of CRC. These findings illustrate that RPPH1 promotes CRC cell metastasis by functioning within cells and changing the tumor microenvironment.

#### 3.2.5. MEG3

Maternally expressed gene 3 (MEG3) is a 1.6 kb lncRNA in the DLK1-Dio3 gene cluster on human chromosome 14q32.3. It has antitumor properties in different cancer cells: breast, liver, glioma, colorectal, cervical, gastric, lung, ovarian, and osteosarcoma [79]. MEG3 expression is significantly reduced in CRC cells compared with normal cells [80]. Its overexpression inhibits the invasion and migration of CRC cells and significantly reduces MMP-2 and MMP-9 expression while increasing tissue inhibitor of metalloproteinase-2 (TIMP-2) expression [80, 81]. In addition, MEG3 overexpression inhibits LPS-induced macrophage apoptosis and secretion of inflammatory factors by inhibiting the activation of the NF-*κ*B signaling pathway [82]. Taken together, this suggests that the immunoregulatory effect of MEG3 is linked with the occurrence and development of CRC and possibly other intestinal diseases.

### 3.3. Exosomal lncRNAs Regulate the Intestinal Mucosal Immune Barrier of CeD

Celiac disease (CeD) is a chronic autoimmune disease that may result from intolerance to gluten ingestion and is prone to occur in people with susceptible genes [83]. It generally manifests as histological lesions of the jejunum, including intestinal villi atrophy and crypt hyperplasia. Clinical manifestations may include diarrhea, abdominal pain, abdominal distension, nausea, vomiting, weight loss, and fatigue [84]. Nucleotide-binding oligomerization domain 1 (NOD1) is an innate immune receptor belonging to the NOD-like receptor (NLR) family and PRRs. It activates multiple proinflammatory pathways by identifying exogenous and endogenous ligands. Gluten peptides are ligands for some innate receptors to activate innate immune reactions including CeD initiation [85, 86]. Mutations in lncRNA HCG14 change NOD1 expression in intestinal epithelial cells leading to CeD [87]. Nevertheless, the precise molecular mechanism underlying the risk variation of HCG14 in the pathogenesis of CeD remains to be clarified.

### 3.4. Other Regulatory Roles of Exosomal lncRNAs in the Intestinal Mucosal Immune Barrier

**Figure 2:**
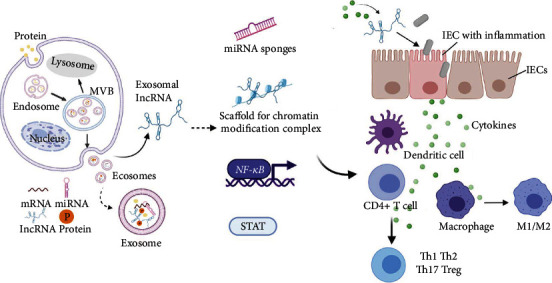
Formation of exosomes and mechanisms of exosomal lncRNA in the intestinal mucosal immune barrier. The formation of the exosome begins at endosomes formation and then maturates to multivesicular bodies (MVBs). MVBs either fuse with lysosomes and are degraded or fuse with the plasma membrane resulting in the release of free exosomes to the extracellular environment. Exosomes may contain various cargoes including proteins, mRNAs, lncRNAs, and miRNAs. Exosomal lncRNAs participate in the intestinal mucosal immune response by influencing the activity of transcription factors or acting as scaffolds for chromatin modification complex, miRNA sponges, translation inhibitors, or mRNA degradation signals. This leads to the regulation of various biological events including the integrity of IECs, polarization of macrophages, differentiation of CD4^+^ T cells, and some inflammatory signaling pathways.

The lncRNA-sekelsky mothers against dpp 3 (lnc-Smad3) recruits histone deacetylase 1 (HDAC1) to *Smad3* promoter which prevents H3K4 methyltransferase Ash1l (absent, small, or homeotic 1-like) from binding to the same region, resulting in inhibition of transforming growth factor-*β*- (TGF-*β*-) induced differentiation of Treg cells [88]. TGF-*β* signal transduction induces phosphorylation, activation, and nuclear translocation of Smad2 and Smad3. The activated Smad complex combines with forkhead box P3 (Foxp3) sites and promotes their expression leading to Treg cell polarization [89, 90]. Ash1l maintains the immune regulatory functions of Treg cells by enhancing the expression of TGF-*β*-induced Smad3 and Foxp3 and promoting the polarization of induced Treg (iTreg) cells. Ash1l-deficient mice are more susceptible to TNBS-induced colitis. This suggests that lnc-Smad3 is involved in intestinal inflammation and that the ASH1L/SMAD3/FOXP3 pathway is involved in human autoimmune pathogenesis by inhibiting the immune regulation of Treg cells [88] (Figure 2 and Table 1).

## 4. Conclusion and Future Outlook

The intestinal mucosal immune barrier participates in humoral immunity and cellular immunity and regulates the intestinal environment. Recent studies have shown that lncRNAs regulate immune cell differentiation and control the inflammatory response by interacting with protein complexes or transcription factors. Exosomal lncRNAs may participate in the regulation of the intestinal mucosal immune barrier and affect the progression of intestinal diseases. For instance, high IFNG-AS1 expression is related to the susceptibility gene of IBD, promotes IFN-*γ* secretion by CD4^+^ T cells, and participates in the development of intestinal mucosal inflammation by regulating the immune function of CD4^+^ T cells.

Some exosomal lncRNAs such as CCAT1 and CCAT2 are highly associated with CRC, but their functions in regulating the intestinal mucosal immune barrier remain unclear. Therefore, further research on these lncRNAs is necessary. There are several exosomal lncRNAs related to an immune response with no relevant studies linking them with the intestinal mucosal immune barrier. It is suggested that future studies should consider including them in the diagnosis and treatment of intestinal diseases.

Studies on the role of exosomal lncRNAs in the intestinal mucosal barrier are in the preliminary stages, and specific mechanisms of their roles in the intestinal mucosal immune system require further study. Exosomal lncRNAs show high organ specificity in blood, urine, saliva, and tumor tissue, have the advantages of being noninvasive, are repeatably detectable, and may be monitored in real-time. Exosomal lncRNAs are expected to provide new ideas and countermeasures for the prevention, diagnosis, and treatment of intestinal diseases through controlling immune mechanisms.

## Figures and Tables

**Table 1:** The potential mechanism of exosomal lncRNAs in the intestinal mucosal immune barrier.

Exosomal lncRNAs	Potential mechanism	References
H19	Antagonizes the negative regulatory factors of intestinal epithelial hyperplasia as an inflammatory lncRNA induced by IL22	[23]
	Participates in inflammatory diseases through VDR signaling	[26]

IFNG-AS1	Increases IFN-*γ* secretion of CD4^+^T cells	[27] [28] [29]

NEAT1	Participates in inflammatory response by regulating intestinal epithelial barrier and exocrine-mediated macrophage polarization	[31]
	Promotes IL-8 expression by relocating SFPQ	[32]
	Participates in TLR2-mediated expression of inflammatory cytokines	[34]

GAS5	Mediates intestinal mucosal by regulating the MMP expression	[44]
	Adjusts the LPS-induced inflammatory destruction by regulating KLF 2 expression and inhibiting the NF-*κ*B pathway	[46]

HIF1-AS2	Inhibits NF-*κ*B signaling pathway activation to protect the immune barrier	[48]

CDKN2B-AS1	Regulates inflammation of UC by sponging miR-195-5p and miR-16-5p and is negatively correlated with levels of inflammatory cytokines	[49] [50]

HOTAIR	Inhibits miR-218 and activates the NF-*κ*B signaling pathway, resulting in the chemical resistance of CRC	[61]
	Acts as a scaffold to form PRC2 complex resulting in CRC development	[62]

CRNDE	Prevents Itch-mediated ubiquitination and degradation of ROR*γ*t to promote Th17 cell differentiation	[71]

MALAT1	Inhibits M2-type macrophage polarization and promotes M1-type macrophage polarization	[73]
	Acts as miR-155 sponge to reprogram DCs into a tolerant phenotype	[75]

RPPH1	Stimulates CRC cell metastasis by promoting exosome-mediated macrophage M2 polarization	[78]

MEG3	Inhibits CRC cell invasion and migration via regulating MMP-2, MMP-9, and TIMP-2	[80, 81]
	Inhibits LPS-induced macrophage apoptosis and secretion of inflammatory factors	[82]

HCG14	Alters NOD1 expression in intestinal cells	[87]

lnc-Smad3	Inhibits Treg cell polarization resulting in T cell-mediated colitis	[88]

## Abbreviations

lncRNAs: Long noncoding RNAs
MVBs: Multivesicular bodies
GALT: Gut-associated lymphoid tissue
PP: Peyer's patch
ILF: Isolated lymphoid follicles
MLN: Mesenteric lymph nodes
IEL: Intraepithelial lymphocyte
LPL: Lamina proprial lymphocyte
M cells: Microfold cells
PAMPs: Pathogen-associated molecular patterns
PRRs: Pathogen recognition receptors
TLRs: Toll-like receptors
VDR: Vitamin D receptor
MAPK: Mitogen-activated protein kinase
IFNG-AS1: Interferon gene antisense RNA-1
STAT: Signal transducer and activator of transcription
T-bet: T-box expressed in T cells
TNBS: 2,4,5-Trinitrobenzenesulphonic acid
HOTAIR: HOX transcript antisense intergenic RNA
PRC2: Polycomb repressive complex 2
LSD1: Lysine-specific histone demethylase 1
EZH: Enhancer of zeste homolog
EED: Embryonic ectoderm development
SUZ12: Suppressor of zeste 12
CRNDE: Colon rectal neoplasia differentially expressed
IECs: Intestinal epithelial cells
ROR*γ*t: Retinoic acid receptor-related orphan receptor gamma-t
DSS: Dextran sulphate sodium
SOCS1: Suppressor of cytokine signaling 1
MALAT1: Metastase-associated lung adenocarcinoma transcript 1
NF-*κ*B: Nuclear factor-kappa B
DC-SIGN: Dendritic cell-specific ICAM-3 grabbing nonintegrin
CCL: Chemokine (C-C motif) ligand
H3K4 methyltransferase: Histone 3 lysine 4 methyltransferase
NEAT1: Nuclear paraspeckle assembly transcript 1
SFPQ: Splicing factor proline/glutamine-rich
CXCL: C-X-C motif chemokine ligand
GAS5: Growth arrest-specific transcript 5
MMPs: Matrix metalloproteinases
Smad: Sekelsky mothers against dpp
HDAC1: Histone deacetylase 1
ASH1L: Absent, small, or homeotic 1-like
Foxp3: Forkhead box P3
EE: Early endosomes
SNP: Single-nucleotide polymorphism
HIF1A-AS2: Hypoxia-inducible factor 1 antisense RNA-2
TAM: Tumor-associated macrophages
RPPH1: Ribonuclease P RNA component H1
MEG3: Maternally expressed gene 3
TIMP-2: Tissue inhibitor of metalloproteinase-2
CeD: Celiac disease
NOD1: Nucleotide-binding oligomerization domain 1
NLR: NOD-like receptor
HnRNPL: Heterogeneous nuclear ribonucleoprotein L
THRIL: TNF-alpha and hnRNPL-related immunoregulatory
TH2-LCR lncRNA: T helper type 2 locus control region lncRNA
RMRP: RNA component of mitochondrial RNA processing endoribonuclease
DDIT4: DNA-Damage Inducible Transcript 4.
